# Modular machine learning for Alzheimer's disease classification from retinal vasculature

**DOI:** 10.1038/s41598-020-80312-2

**Published:** 2021-01-08

**Authors:** Jianqiao Tian, Glenn Smith, Han Guo, Boya Liu, Zehua Pan, Zijie Wang, Shuangyu Xiong, Ruogu Fang

**Affiliations:** 1 J. Crayton Pruitt Family Department of Biomedical Engineering, University of Florida, Gainesville, FL 32611 USA; 2 Department of Clinical and Health Psychology, University of Florida, Gainesville, FL 32611 USA; 3 College of Electrical Engineering, Zhejiang University, Hangzhou, 310000 China; 4 School of Information and Telecommunication Engineering, Beijing University of Posts & Telecommunications, Beijing, 100876 China; 5 School of Electrical and Information Engineering, Beijing Jiaotong University, Beijing, 100044 China; 6 School of Mathematical Science, East China Normal University, Shanghai, 200062 China; 7 Department of Data Science, East China Normal University, Shanghai, 200062 China; 8 Department of Electrical and Computer Engineering, University of Florida, Gainesville, FL 32611 USA; 9 Center for Cognitive Aging and Memory, McKnight Brain Institute, University of Florida, Gainesville, FL 32610 USA

**Keywords:** Biomedical engineering, Diagnostic markers, Dementia, Alzheimer's disease

## Abstract

Alzheimer's disease is the leading cause of dementia. The long progression period in Alzheimer's disease provides a possibility for patients to get early treatment by having routine screenings. However, current clinical diagnostic imaging tools do not meet the specific requirements for screening procedures due to high cost and limited availability. In this work, we took the initiative to evaluate the retina, especially the retinal vasculature, as an alternative for conducting screenings for dementia patients caused by Alzheimer's disease. Highly modular machine learning techniques were employed throughout the whole pipeline. Utilizing data from the UK Biobank, the pipeline achieved an average classification accuracy of 82.44%. Besides the high classification accuracy, we also added a saliency analysis to strengthen this pipeline's interpretability. The saliency analysis indicated that within retinal images, small vessels carry more information for diagnosing Alzheimer's diseases, which aligns with related studies.

## Introduction

Alzheimer's disease is the leading neurodegenerative disease, estimated to affect roughly 13–16 million patients by 2050^1^. Similar to other neurodegenerative diseases, neural damage caused by Alzheimer's disease is often irreversible. Current clinical treatment strategies focus on slowing down the accumulation of pathology, not restoration of neurological function. Therefore, routine screening and early diagnosis are critical in maximizing treatment benefits and preserving neural functions.

The current gold standard for diagnosing Alzheimer's disease is based on detecting amyloid-beta abnormality. This diagnosing evaluation can be done cerebrospinal fluid (CSF)^2–4^, but this invasive process introduces health risks to patients. Non-invasive brain imaging, such as positron emission tomography (PET) combined with structural MRI imaging, has been evaluated as an alternative to CSF for accurate and sensitive diagnosis of Alzheimer's disease^5^. These techniques can capture multiple characteristics of the brain, such as the structural changes in the brain^6^, amyloid-beta density, or neural tissue metabolism activity. Nevertheless, these neuroimaging modalities still cannot fulfill the demand for timely Alzheimer's disease screening because they are relatively expensive and have limited accessibility. Amyloid PET typically costs three thousand to four thousand US dollars out of patients' pockets^7^.

Alternatively, the retina presents a readily accessible window for extracting potential biomarkers of Alzheimer's disease. The retina is the only component of the nervous system that can be *directly* observed in vivo. Recent studies demonstrate that retinal fundus images display pathological features associated with the early stage of neurodegeneration diseases. For example, the thickness of the retinal nerve fiber layer^8^ and visual acuity^9–11^ are associated with early-stage cognitive impairment^12^. In pre-symptomatic transgenic Alzheimer's disease mice, amyloid-beta plaques have been observed to emerge 2.5 months earlier on the retina than in the brain^13^.

Among these retinal biomarkers, retinal vasculature frequently has a marked association with Alzheimer's disease. Abnormal narrowing in retinal venous blood column diameter and decrease blood flows are reported to be correlated in patients with Alzheimer's disease^14^, potentially explaining the observed reduced retinal oxygen metabolism rate in mild cognitive impairment subjects group^15^. A sparse fundus vascular network with decreased vascular fractal dimensions has a strong association with dementia^16^. The retinal neurovascular coupling is also found to be impaired in subjects with Alzheimer's disease compared to healthy aging^17^. The emerging evidence drives us to assume that retinal vasculature could potentially be useful for early detection of Alzheimer's disease. However, previous retinal imaging studies are limited by two common drawbacks. First, they involve a relatively high level of manual labeling, such as segmenting and measuring the thickness of the retinal nerve fiber layer. This manual labeling requirment potentially introduces human error. Second, the investigated features were generated based on fixed hand-picked rules and contained less adaptability. Recent studies of automated machine learning applied to retinal fundus images have shown promising results in detecting several diseases, including glaucoma^18^, diabetic retinopathy^19^, anemia^20^, choroidal neovascularization, central serous chorioretinopathy, vitreomacular traction syndrome^21^, as well as identifying cardiovascular risk factors such as gender, smoking status, systolic blood pressure, and body mass index^22^. We hypothesize that machine learning techniques are capable of recognizing retinal vascular features associated with Alzheimer's disease, which are difficult for even human experts to identify. Specifically, we have developed a multi-stage machine learning pipeline to explore this hypothesis's feasibility in a highly automated fashion. A fully automated machine learning framework can substantially reduce the need for manual labeling, thus enables future studies to have larger scales.

## Result

This study was conducted on a recently released open-access database, the UK Biobank^23^, which is a prospective, ongoing nation-wide cohort following ~ 500,000 individuals with ages ranging from 40 to 69 (at the time of initial enrollment) across the United Kingdom. This unprecedented database has 7,562 fields, including imaging, genetics, clinical, and environmental exposure data. The rich retinal imaging and diagnostic data in UK Biobank enable us to study retinal biomarkers for Alzheimer's disease through automated machine learning.

The UK Biobank provides an opportunity to develop and validate methods for Alzheimer's disease detection from the general population, in contrast to existing research built on cohorts of patients with specific diseases. The quality of image data from the UK Biobank is also more consistent with clinical data collected in everyday healthcare practice, compared to high-quality research-oriented data collected in existing studies. For example, the retinal fundus images in UK Biobank were collected as a viewfinder for follow-up optical coherence tomography (OCT)^24^, resulting in fundus images with substantially varying quality. We decided to evaluate our method's performance on a real-world, clinically collected database rather than high-quality research-oriented databases since we aim to estimate how much impact our proposed method could bring to the clinical community. Another advantage of the UK Biobank to address this research question is the quality of dementia labels. We used the “Dementia Outcome: Alzheimer’s disease” label from UK Biobank Electronic Health Records data to identify subjects with definite Alzheimer’s Disease Diagnosis, which is based on a comprehensive evaluation procedure instead of a single test-based label, making this AD diagnosis more reliable. In sum, the UK Biobank enables the development of an automated machine learning method to classify Alzheimer's disease dementia, distinct from healthy aging, by identifying retinal changes associated with Alzheimer's disease using clinical-level data collected from the general population. In other words, if successful, our methods would be immediately and highly generalizable.

### We utilized a multi-task pipeline that can be deployed modularly

**Figure 1:**
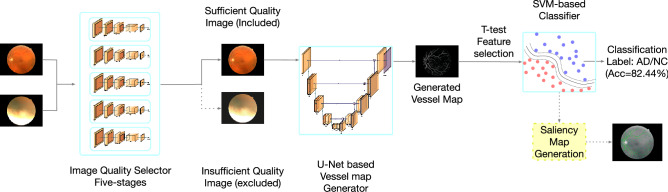
The overall pipeline. The whole pipeline contains three major stages: image quality selection, vessel map segmentation, and SVM-based classifier.

**Figure 2:**
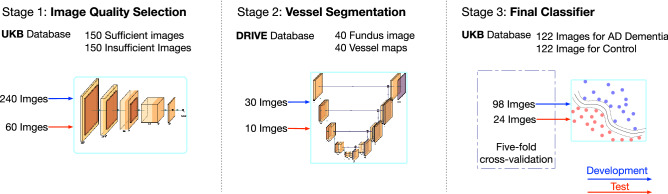
Each stage can be trained separately. Compared with end-to-end structure, this multi-stage pipeline has an advantage as being modular where each stage can be trained independently with various databases. This advantage could be highly valuable when this pipeline needs to be applied to a new database and only one or two stage needs to be retrained.

The majority of popular machine-learning-based studies generally achieved their tasks by end-to-end network architecture. Instead, we adopted a multi-stage architecture in this study. Specifically, the overall pipeline includes three cascaded steps: image quality selection, vessel map generation, and Alzheimer's disease classification, as shown in Fig. 1. Two major reasons motivated us to adopt this multi-stage design. First, having an independent performance at each step increases our control over each specific stage, i.e., we can validate and adjust each step separately. As a benefit, such a pipeline has strong adaptability. In this study, the UK Biobank database does not have all the labels required by all the stages in the whole pipeline. With this multi-stage design, we managed to train each stage separately with other databases but tested the whole pipeline on the UK Biobank database, as shown in Fig. 2. For new datasets, we can also transfer our trained machine by only retraining part of the pipeline with easier-to-obtain labels. Second, the multi-stage pipeline improves overall interpretability. Each step has an explicit purpose, so we can understand how each step contributes to the final classification decision. Domain knowledge is easier to embed within such a structure as well.

**Figure 3:**
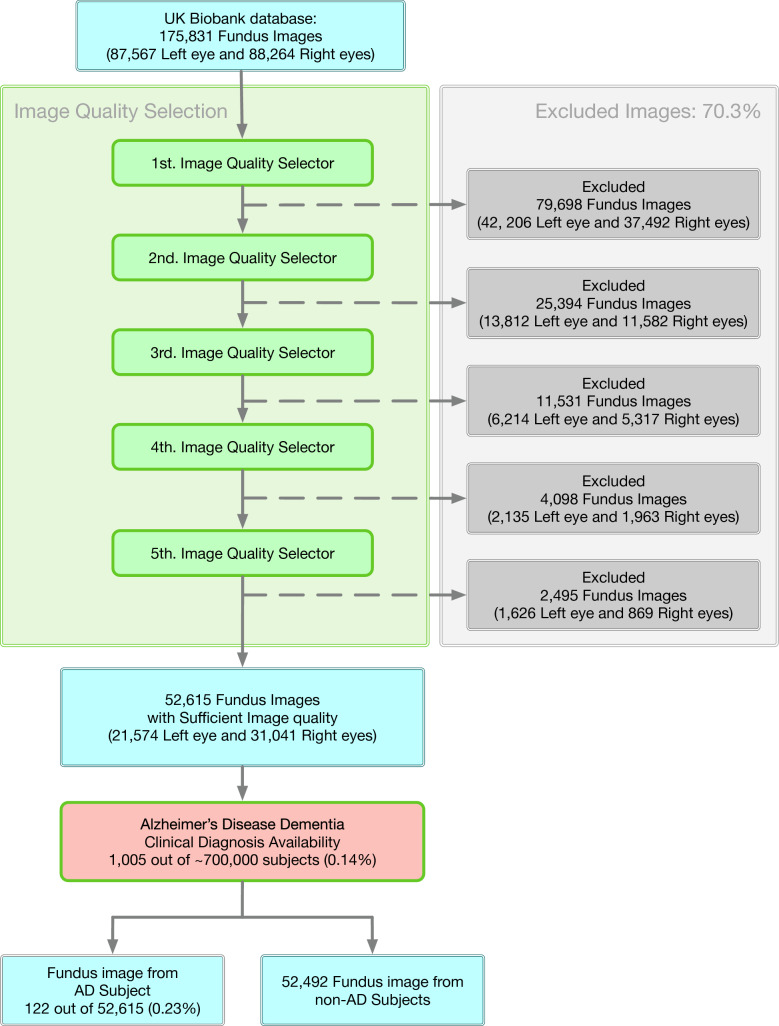
Database selection rate flow. The five selectors are cascaded, meaning one image is considered to be a sufficient image only if it passes all five selectors. We have a database containing 122 fundus images from 87 AD subjects.

**Figure 4:**
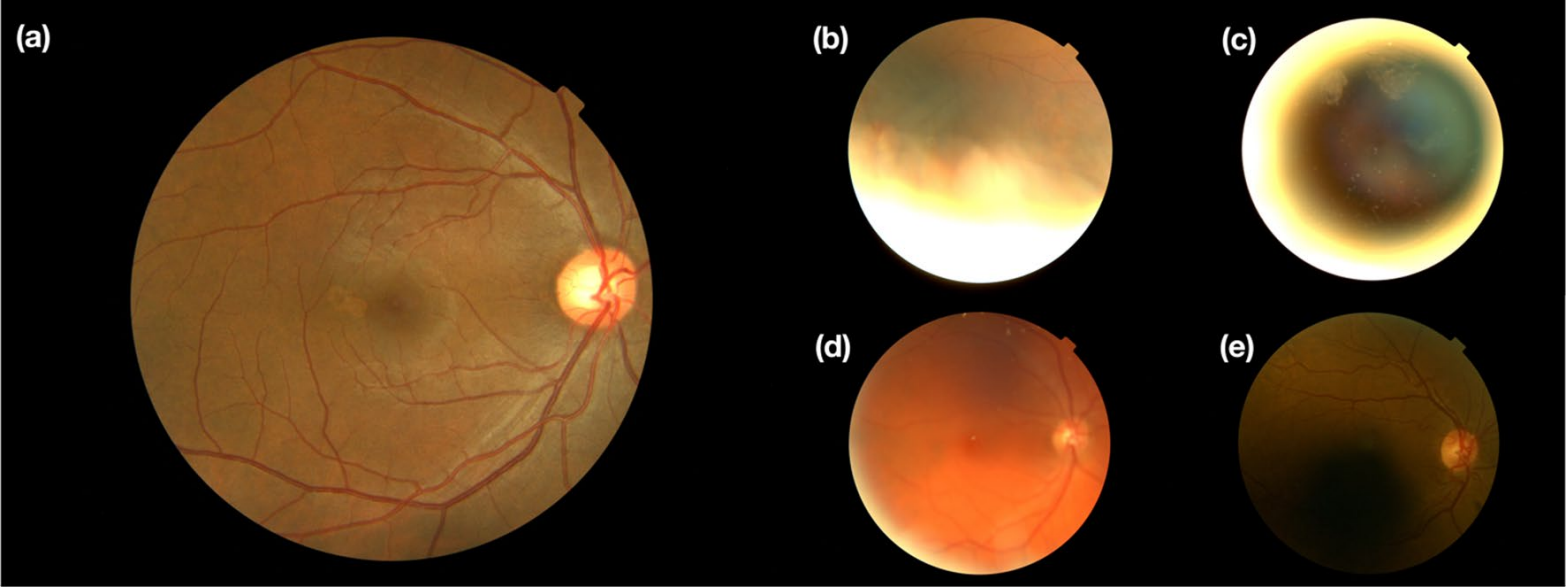
Demonstration of selected images. (**a**) Ideal image quality. (**b**) Insufficient image quality with artifacts. (**c**) Insufficient image quality with bad composition. (**d**) Insufficient image quality due to being out-of-focus. (**e**) Insufficient image quality with unbalanced and insufficient illumination.

When dealing with databases collected from clinical practice, one of the foremost limitations is inconsistent image quality. In this current study, fundus image quality was evaluated for image composition, exposure/contrast, artifacts, and sharpness/focus^25^. Selecting good images out of the whole database is solved as an image classification task^25^ in this study. Specifically, we simulated a "multi-reviewer" decision-making mechanism by training five separate image quality classification networks with identical hyper-parameters, structure, and datasets but with different initialization conditions. Each image had to pass all five classifiers unanimously to be included in our sufficient image datasets. This is a much stricter selection compared with "majority-vote." We employed this strict standard because we anticipated that insufficient images would introduce more potentially non-pathological image features, such as artifacts, misguide the final classification and decrease final performance and resulting interpretability. At the time of data acquisition, the UK Biobank had 87,567 left fundus images and 88,264 right fundus images. After the image quality selection, 21,547 left fundus images, and 31,041 right fundus images were extracted into our sufficient image database. Figure 3 illustrates the selection rate through each step of the image quality selection process. Figure 4 provides some examples of Sufficient and Insufficient fundus images classified by this quality selection process. After the fundus image quality control (Method), 122 sufficient fundus images from 87 Alzheimer's disease patients were found to have a valid clinical diagnosis of Alzheimer's disease dementia, i.e., the most advanced stage of Alzheimer’s disease. The cohort characteristics were presented in Table 1.

**Table 1:** Cohort characteristics of all extracted groups.

	Number of subjects (male/female)	Number of images (left eye/right eye)	Age at recruitment (mean/Std.)
AD Dementia Group	87 (46/41)	122 (77/45)	65.17 (4.16)
Five Control Groups	87 (46/41)	122 (77/45)	65.17 (4.16)

**Figure 5:**
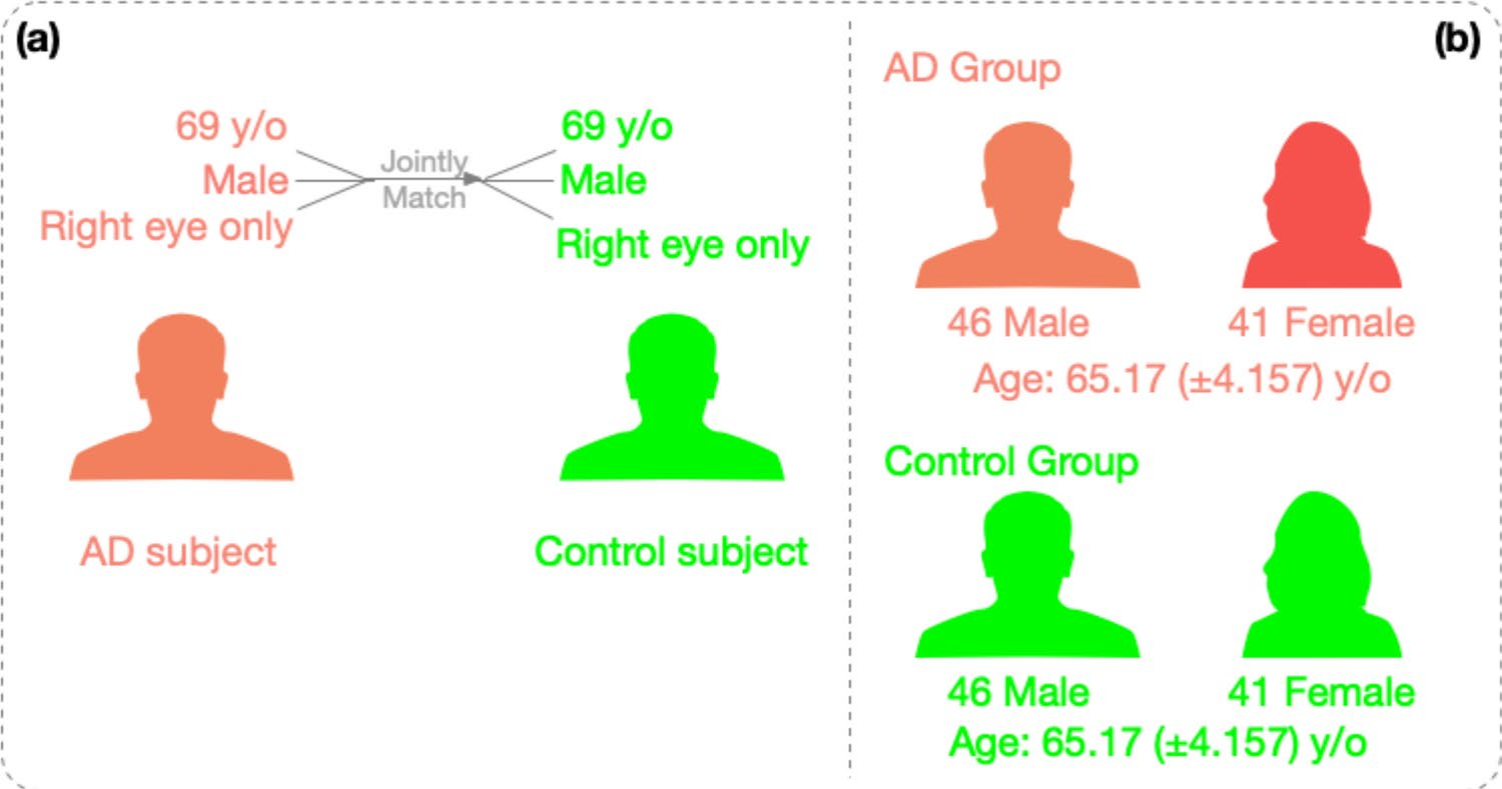
Control subjects were matched at the individual level. (**a**) For each AD subject, the matched healthy control subject was found with the identical combination of gender and age. (**b**) As a result, the control group and the AD group have identical distributions of gender and age.

The matching of healthy control subjects was achieved at an individual-subject level. For each subject in the Alzheimer’s disease group, the healthy counterpart was found by matching both gender and age (when fundus images were taken). For images that belong to the same subject with Alzheimer’s Disease, their counterparts will be extracted from the same subject as well. This matching standard was proposed so that the subject-dependent bias will be eliminated as much as possible. As a result, we obtained two datasets highly controlled for demographical information, as shown in Fig. 5, for both Alzheimer’s disease group and healthy control groups. This study has redundently extracted five control groups for Alzheimer’s disease dementia group to evaluate our pipeline’s performance. For every subject in the AD group, we extracted five different control subjects with the same age, gender, and image for the same eye side as the AD subject, following the same subject matching procedure. These five subjects were then randomly assigned into five control groups separately.

### The proposed pipeline is effective in distinguishing Alzheimer's disease from healthy control images

We evaluated the binary classification performance in terms of sensitivity, specificity, classification accuracy, and F-1 score. These performance metrics were calculated at the image level instead of the subject level since not all selected subjects have both eyes included. To maximally leverage the current database, we employed a nested five-fold cross-validation strategy (Method). The performance is summarized in Table 2. To further boost the performance, we employed a T-test (*p*-value = 0.01) to select the pixels that carry the most information to distinguish between two groups. With this feature selection, the performance classification accuracy was improved from 68.2% to 82.4%. Table 3 reports the detailed performance with t-test feature selection. Overall, the result is highly consistent across all healthy control groups, indicating the reported performance doesn't rely on any specific healthy control group. The effectiveness of such a pipeline has been demonstrated and proven. The performance is consistent as well based on the small variance measured from five-fold cross-validation.

**Table 2:** Performance comparison of the overall classification for AD versus normal controls (NC)* ***without feature selection***.

Classification Groups	Accuracy (mean/std)	Precision(PPV) (mean/std)	NPV (mean/std)	Specificity (TNR) (mean/std)	Sensitivity (TPR) (mean/std)	F1-Score (mean/std)
AD group versus 1st normal control group	**0.734** (0.062)	**0.778** (0.048)	**0.694** (0.077)	**0.808** (0.069)	0.648 (0.110)	**0.702** (0.078)
AD group versus 2nd normal control group	0.674 (0.072)	0.692 (0.084)	*0.658* (0.064)	0.758 (0.095)	*0.584* (0.143)	0.632 (0.095)
AD group versus 3rd normal control group	0.680 (0.090)	0.684 (0.091)	0.674 (0.077)	*0.678* (0.118)	**0.678** (0.061)	0.680 (0.073)
AD group versus 4th normal control group	0.668 (0.074)	0.666 (0.078)	0.664 (0.084)	0.706 (0.079)	0.624 (0.067)	0.644 (0.073)
AD group versus 5th normal control group	*0.654* (0.085)	*0.658* (0.078)	0.664 (0.088)	0.686 (0.074)	0.618 (0.176)	*0.630* (0.130)
Average over five datasets	0.682	0.6956	0.6708	0.7272	0.6304	0.6576

*The mean and standard deviation values were extracted from five-fold cross validations.

**Bold** font represents the most optimal performance for each measurement among five-fold cross validations. *Italic* font represents the least optimal performance.

Abbreviation: AD = dementia caused by Alzheimer’s disease, NC = normal control, PPV = positive predictive value, NPV = negative predictive value, TNR = true negative rate, TPR = true positive rate.

**Table 3:** Performance comparison of the overall classification for AD versus normal controls (NC)* ***with feature selection***.

Classification Groups	Accuracy (mean/std)	Precision(PPV) (mean/std)	NPV (mean/std)	Specificity (TNR) (mean/std)	Sensitivity (TPR) (mean/std)	F1-Score (mean/std)
AD group versus 1st normal control group	0.8216 (0.0136)	0.8504 (0.0186)	0.7977 (0.0144)	0.8623 (0.0200)	0.7811 (0.0189)	0.8142 (0.0143)
AD group versus 2nd normal control group	0.8280 (0.0142)	0.8316 (0.0115)	0.7883 (0.0191)	0.8424 (0.0119)	*0.7730* (0.0256)	0.8010 (0.0167)
AD group versus 3rd normal control group	0.8300 (0.0110)	**0.8561** (0.0121)	0.8079 (0.0177)	**0.8664** (0.0145)	0.7934 (0.0250)	0.8233 (0.0134)
AD group versus 4th normal control group	**0.8426** (0.0129)	0.8432 (0.0105)	**0.8423** (0.0170)	0.8434 (0.0105)	**0.8418** (0.0193)	**0.8424** (0.0137)
AD group versus 5th normal control group	*0.7999* (0.0130)	*0.8172* (0.0100)	*0.7851* (0.0197)	*0.8270* (0.0119)	*0.7730* (0.0276)	*0.7942* (0.0161)
Average over five datasets	0.82442	0.8397	0.80426	0.8483	0.79246	0.81502

*The mean and standard deviation values were extracted from five-fold cross validations.

**Bold** font represents the most optimal performance for each measurement among five-fold cross validations. *Italic* font represents the least optimal performance.

Abbreviation: AD = dementia caused by Alzheimer’s disease, NC = normal control, PPV = positive predictive value, NPV = negative predictive value, TNR = true negative rate, TPR = true positive rate.

### The effectiveness of the pipeline is not data reliant, as validated with redundant experiments

Small databases are often viewed as a weakness in machine learning-based studies. One major reason is small databases can easily limit the generality of the trained machine model since a small database is less likely to include all the variance and miss relatively rare data points. In addition, the trained machine model can become more data reliant if trained on a smaller database. Data-reliance refers to a situation where the machine learning model overfits the training dataset, and the classification was not actually made based on the general image features, but rather memorizing the data or co-existent features. One good example is disease classification from medical images collected from multiple sites, where each site uses different scanners with distinct image formats. When certain study sites have a strong association with a specific disease, a high classification accuracy could be achieved by distinguishing image format on the data collection site, instead of pathological features.

In this current study drawing on a database with over 500,000 individuals, even we were limited with the number of fundus images for Alzheimer's disease patients, however, we had a wide choice of healthy control subjects. To remedy the small-dataset drawback, we designed more experiments to test the classifier's performance repeatedly. We defined multiple healthy control groups to repeat the Alzheimer's disease versus healthy control classification. By repeating the Alzheimer's disease group versus multiple healthy control groups, we could test if the classification performance was influenced by one specific healthy control group.

### The saliency map shows interpretable features from the trained machine learning model

**Figure 6:**
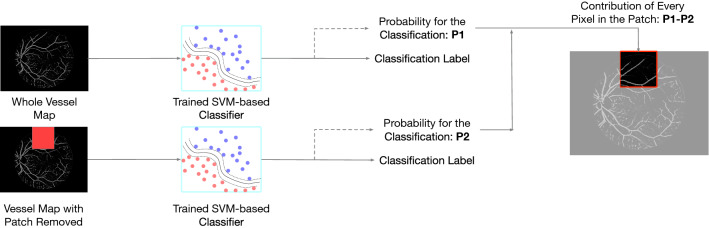
Occlusion tests were used to generate saliency maps in this study. The occlusion test measures the importance of a certain area for the predicted label by measuring the probabilities of the target classification label with and without this area in the image (Method).

**Figure 7:**
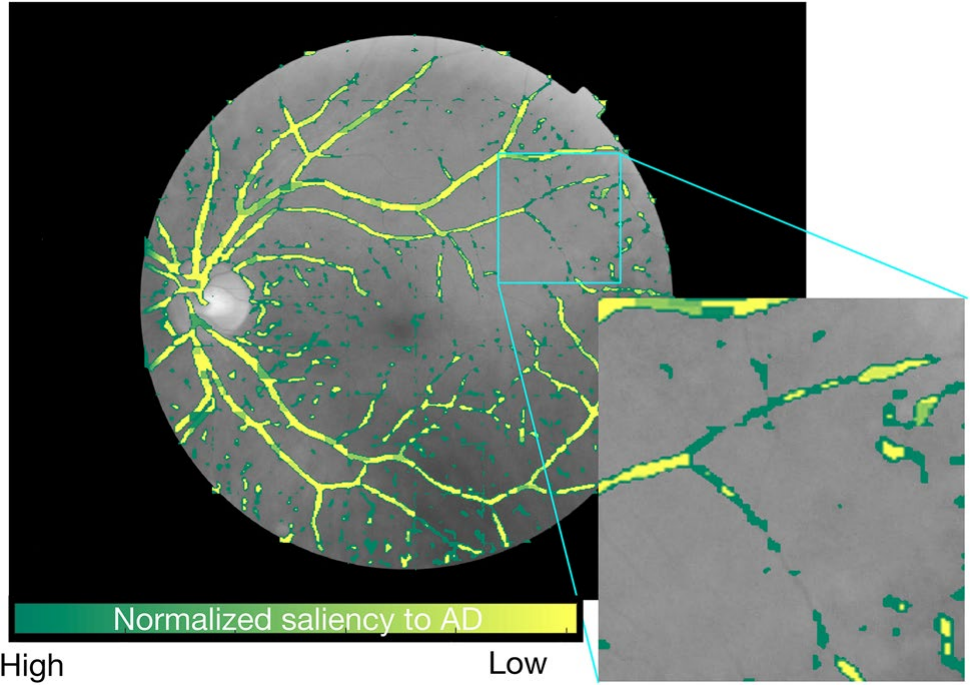
Generated saliency map for an image belongs to an AD subject and corrected classified. Green pixels are more salient for classifying AD, while yellow pixels do not significantly contribute to this classification result. According to this saliency map, small vessels and capillary vessels are more important in determining whether this image belongs to an AD subject.

In addition to the blind tests, we employed another approach to verify if our trained machined learning model had captured image features of anatomical or pathological importance. We used the occlusion test ^26^ to extract and evaluate the varying importance of each part of vessel maps. The occlusion test evaluates the importance of a certain area by measuring the variation of prediction likelihood with and without such area in the original image (Method). This test procedure can be described in Fig. 6. Figure 7 illustrates one example of such a saliency map. This saliency map reflects the importance of different regions from the vessel maps at various levels, from pixel level to larger 32-by-32 patch area. A general observation we can make through these saliency maps is that small vessels contribute more than major vessels for Alzheimer's disease classification. Our observation aligns with previous studies regarding the vessel map features in Alzheimer's progression^16^. During the process of vessel diameter narrowing^14^ and venular degeneration^27^ associated with Alzheimer's disease, small vessels are more vulnerable to morphological changes. Therefore, it is understandable that our trained machine gives higher attention to small vessel areas. Meanwhile, we observed that even within a small neighborhood, the importance varies greatly on the individual pixel as well. This is a benefit of a machine learning approach because the network can comprehend data at multiple levels, including pixel level, which humans cannot achieve.

## Discussion

Emerging evidence suggests that Alzheimer's disease has a pre-symptomatic period that can be 40–50 years long since PSEN1 E280A mutation carriers are showing cerebral spinal fluid abnormality as early as in their 20s^28,29^. Such a long pre-symptomatic period motivates efforts to find a potential in vivo image biomarker that is suitable for timely routine screening of Alzheimer's disease. This study focuses on the feasibility of identifying potential links between the retina vasculature and Alzheimer's disease using machine learning techniques. Based on the results we obtained from the present experiments, the retina does seem to be a strong and effective candidate site as a potential biomarker of Alzheimer's disease. Previous work has attempted to uncover a connection between Alzheimer's disease and the retina^14,30–35^. Although highly innovative and inspiring, previous research had two major limitations. First, the methods typically required intense manual measurement of biomarkers. Second, conventional "group-level" retinal image data analysis techniques can only separate group-level averages and not perform individualized predictions. This limitation substantially impedes its clinical translation. Combining retinal image analysis with machine learning techniques overcomes both of these limitations. First, this proposed machine learning pipeline is capable of achieving multiple tasks, such as image quality control, vessel map generation, and final classification, in a highly automated fashion. Besides the reduction of manual labor, having a highly automated classification algorithm also helps to eliminate potential human error and bias. Second, this proposed machine learning-based algorithm can bring out a clear classification result, along with an interpretable saliency map that explains which areas of the vessel maps were given special consideration when making a classification decision. This additional interpretability may help focus on pathophysiology research. The saliency map findings raise the question of why these specific vessel regions are salient. What is the pathological process associated with Alzheimer’s disease that is occurring specifically in these small vessel regions? An additional benefit of this work is the generality. Using a population-based sample, we trained our algorithm separately for each task stage, utilizing more than one data source. For machine learning techniques, when the developmental datasets and validation testing datasets are collected from different sources, the domain barrier existent between the two data sources will generally decrease the overall performance and limit the model's generalizability. However, in this current study, we demonstrated that even if we used different databases in the development stage from the validation stage, the overall pipeline still classifies Alzheimer's disease from healthy controls. Specifically, we used the DRIVE database to train vessel segmentation networks (Method) since the UK Biobank database does not provide vessel maps for training purposes. Then the validation stage was carried out solely on the UK Biobank database. The overall performance indicates current pipeline design overcomes the database domain barrier and achieves higher generality.

The human interpretable biomarker features are expressed in the form of the saliency map (Method). One general observation from these saliency maps is that the small vessel's morphology is critical in making the classification decision, in comparison to major vessels. We have found this observation strongly aligns with other findings, such as cerebral vascular changes related to Alzheimer's disease and cognitive impairment^36^. The co-existence between the constriction of small arteries and arterioles and Alzheimer’s disease has been investigated as a major perspective of neurovascular dysfunction^37^. This neurovascular dysfunction could be both the cause and result of the structural degeneration and functional impairment associated with Alzheimer’s disease. The loss of structural and functional connectivity could be amyloid-dependent as well as amyloid-independent pathways. Therefore, for AD patients who do not show significant amyloid deposits, the small vessel changes can still be a valid diagnosis biomarker. Furthermore, the accumulation of toxic amyloid-beta in the vessel has been suspected of causing dysfunction in the blood–brain barrier in aged subjects^38^. In addition to cognitive impairment, 84% of patients with Alzheimer's disease have also reported morphological substrates of cerebrovascular diseases^39^. Venular degeneration was found to be closely associated with Alzheimer's disease in a transgenic animal study^27^. Moreover, retinal venular vessels are related to multiple diseases such as diabetes^40^, aging^41^, and especially neurodegenerative diseases^42^. On the other hand, machine learning methods are also capable of finding deeper level features. We have found that even in a very small neighborhood on the major vessels, changes associated with individual pixels can be important in making the overall classification conclusion. The machine learning-based technique is capable of making the final decision by considering all pixels. Compared with human experts, such a unique property of the machine learning-based method sheds light on finding retinal imaging biomarkers at a deeper level.

However, it should be clarified that this work only serves as an explorative study, even if it does show promising results. This proposed work has its major limitations introduced by the used database. The most fundamental limitation exists in the population distribution of Alzheimer's disease. Within the 502,505 subjects we extracted from UK Biobank, 40% of them are older than 60-year-old at recruitment (201,002 subjects). According to general population studies^43^, about 6% of people of age above 60 are expected to have dementia with Alzheimer’s, accounting for approximately 70% of cases^44^. Therefore, the expected number of Alzheimer's disease patients in the UK Biobank is around 8,400. However, there are only 1,005 subjects diagnosed and documented with Alzheimer's disease in the whole UK Biobank. The number of patients who had fundus images with sufficient quality further dramatically decreased the sample size. Two potential factors could help explain this gap. On the one hand, UK Biobank is known to have a healthier population compared with the general population in the UK^45^. For example, lung cancer in the UK Biobank is 10%-20% lower than the general population. On the other hand, we cannot exclude the possibility that a substantial amount of Alzheimer's disease went undetected or undocumented in the UK Biobank, including some of our controls. With five control datasets that were randomly extracted, following the same individual match principle, we aimed to reduce the influence of this undetected subject group.

Also, unlike the majority of machine learning studies that used tens of thousands of data samples for development and validation, the database in this work is relatively small, due to the requirement of sufficient-quality retinal images for subjects with Alzheimer's disease. A small database is the main reason that we cannot easily apply deep learning architecture in an end-to-end fashion in this current work. Even if we carefully designed multiple sets of control experiments, following the strict subject matching protocol, to strengthen our conclusion by eliminating data reliance as much as possible, our conclusions would require future investigation on its generality in a larger population/cohort. Our reported performance from multisets of control experiments is only essential but not sufficient for claiming the reported result doesn't contain data-reliance since we cannot ideally include all possible control parameters.

In summary, this work demonstrates that with proper development, the machine learning-based technique is efficient and effective in discovering potential image biomarkers for Alzheimer's disease from retinal vasculature systems., This study shows that retinal vasculature analysis using machine learning could be an effective and cost-efficient approach for Alzheimer's disease screening.

## Method

### Study participating database

This study was conducted primarily with the UK Biobank database, especially for the validation stage. Cross-disease labels have a high value in studying the correlation between target diseases. At the time when this work was conducted, there were 87,567 left fundus images and 88,264 right fundus images available. After a strict quality selection process, 24% of left fundus (O.S.) images and 36% of right fundus (O.D.) images passed the quality selection (compared with 88% in^22^).

In the UK Biobank, the label of Alzheimer's disease dementia was based on ICD codes found in hospital admission and death records, which indicated a definitively clinical diagnosis for dementia caused by Alzheimer's disease (Data-field 42021)^46^.

Subjects with at least one high-quality retinal image and a positive Alzheimer's disease label were included in the Alzheimer's disease group of the final dataset. In our case, we extracted 122 fundus images, belonging to 87 subjects from the Alzheimer's disease group.

### Image quality selection

One major challenge in conducting data-driven research with a large-scale non-disease specific database is to deal with data inconsistency introduced by the data collection protocols. Specifically, the fundus images in UK Biobank have a substantial image quality variability, since fundus images are collected as the viewfinder for OCT images, instead of being a primary data field for diagnosis.

Prior to building the machine learning model for Alzheimer's disease versus healthy controls classification, it is critical to have consistently high-quality fundus images for training and testing the classifier. Otherwise, the classification results could be negatively impacted by the low image quality. Image quality could be influenced by many other factors than Alzheimer's disease, such as age, scanning site, OCT/fundus scanning protocols. Without controlling the image quality, the classifier could mistakenly classify the disease based on the quality differences. For example, studies show that elderly patients tend to have lower-quality fundus images due to the low transparency of their lens^47^.

We used a multi-phase CNN-based image classification network to perform image quality selection. The factors leading to poor image quality are over- or under-exposure, out of focus, faulty composition, and artifacts. Images with any of the above issues will be classified as having "insufficient quality." Similar criteria have been previously used for assessing fundus image quality with machine learning techniques before^25^. Following this rating standard, we establish a medium-size database with 150 images with sufficient quality and 150 images with insufficient quality to train five independent networks with the same structure and hyper-parameter but different random initialization. For each image, the five networks return independent classification labels. The image will then be classified as with sufficient quality only if all five independent classifier results agree to be sufficient.

Noteworthily, the definition of sufficient image quality is a subjective topic. Standards vary dependent on clinical demands. Our study applies a relatively stringent standard in image quality selection due to the following machine learning module for Alzheimer's disease classification. Low-quality images input into the machine learning pipeline could generate unexplainable results at the end of the process. In the following procedure, we will only use images with sufficient quality for further development and validation.

### Vessel map generation

Segmenting vessel maps from fundus images is a typical image segmentation task and can be confidently achieved by using U-net^48,49^. During the development process, we train a vessel segmentation deep learning model on the DRIVE database^50^ and evaluate it on the UK Biobank dataset. Indeed, it is a less common practice in developing a machine learning-based algorithm to train on one database and apply it to a different database. The reason for our current design is based on one important reason: To test the generality of the proposed method. When the trained algorithm was present to a new database, it can be expected that this trained algorithm may not work well without proper domain adaptation. However, in the clinical setting, it is almost unavoidable that data were collected from different sites use different devices, introducing device-based domain variation into the database. The current development/validation dataset configuration enables us to discuss how robust this proposed pipeline works when facing an unseen database.

### Alzheimer's disease classification

The Alzheimer's disease classifier model in this study is a binary support vector machine (SVM)-based classifier. A T-test feature selection procedure was employed before inputting the generated vessel maps into the classifier, *p*-value thresholded at 0.01. The feature selection was conducted only on the training folds, so that the testing fold is not leaked to the feature selection or classifier in any way. Specifically, the t-value of each pixel at (x,y) can be calculated as:

$$t_{{\left( {x,y} \right)}} = \frac{{\overline{I}_{{\left( {x,y,AD} \right)}} - \overline{I}_{{\left( {x,y,CN} \right)}} }}{{\sqrt {\left( {\frac{{\sigma^{2}_{{\left( {x,y,AD} \right)}} }}{{n_{AD} }} + \frac{{\sigma^{2}_{{\left( {x,y,CN} \right)}} }}{{n_{CN} }}} \right)} }}$$

where $\overline{I}_{{\left( {x,y,class} \right)}}$ denotes the averaged intensity at pixel (x,y) for all images in the *class* group (either AD or CN) in training dataset; $\sigma^{2}_{{\left( {x,y,class} \right)}}$ denotes the standard deviation of the intensity at pixel (x,y) for all *class* group (either AD or CN) images in training dataset; $n_{class}$ denotes the number of images in *class* group (either AD or CN) images in the training dataset.

The t-value of each pixel at (x,y) will then be compared with a threshold t-valve with *p* = 0.01 (from a pre-defined lookup table) to verify its statistical significance in differentiating AD from NC. The pixel will be selected as a key feature if its t-value is deemed as statistically significant.

The input to SVM is a vector of selected features from the T-test, and the output is a binary scalar, representing if the fundus image came from a subject with Alzheimer's disease or healthy control. Based on empirical results, the Gaussian radial basis function (RBF) was chosen as the SVM kernel. We applied a nested five-fold cross-validation protocol for developing and testing the overall classification performance based on the whole 122 vessel map images. The entire dataset was divided into five-folds, with four external folds for training and validation, and one external fold left for testing in each round. Another internal five-fold cross-validation is performed inside the training and validation data to optimize hyper-parameters in RBF SVM using a grid search. The optimal hyper-parameters were used to train an SVM model that was tested on the one external fold left out. This process is done five times, so each external fold was used as test data once. The overall performance was reported as the average performance on all folds.

### Saliency maps

Machine learning-based techniques generally have a limitation in a lack of interpretability. To increase the interpretability, we proposed to obtain saliency maps by using occlusion tests^26^ to visualize the contribution of different parts of the vascular system to the machine learning prediction. Specifically, we defined a set of patches with various sizes, ranging from 1-by-1 (pixel), 2-by-2, 4-by-4, to 8-by-8. Then, for each patch size, we move the patch in a sliding fashion over the entire image, setting the pixels overlapping with the patch to zero (blackout), and calculate the importance of regions covered by the patch as the change in classification probability confidence for the output label. The more information one area carries, the larger difference between the original image and image with this area occluded. And if the area is not meaningful for the classification at all, such as pixels of backgrounds and artifacts, occluding this area would have no impact on outcome probability.

For each window scale, the saliency at each window location was normalized into the range of $\left( {0,1} \right)$ and assigned to the central pixel in that window. Once collusion tests were accomplished at all window levels, each pixel’s final saliency value will be the average saliency values from all window scales, which is visualized in the final saliency map.

Eventually, the saliency scores at each pixel location across all patch scales were summarized and normalized to generate saliency maps, where the intensity of each pixel represents its importance.
